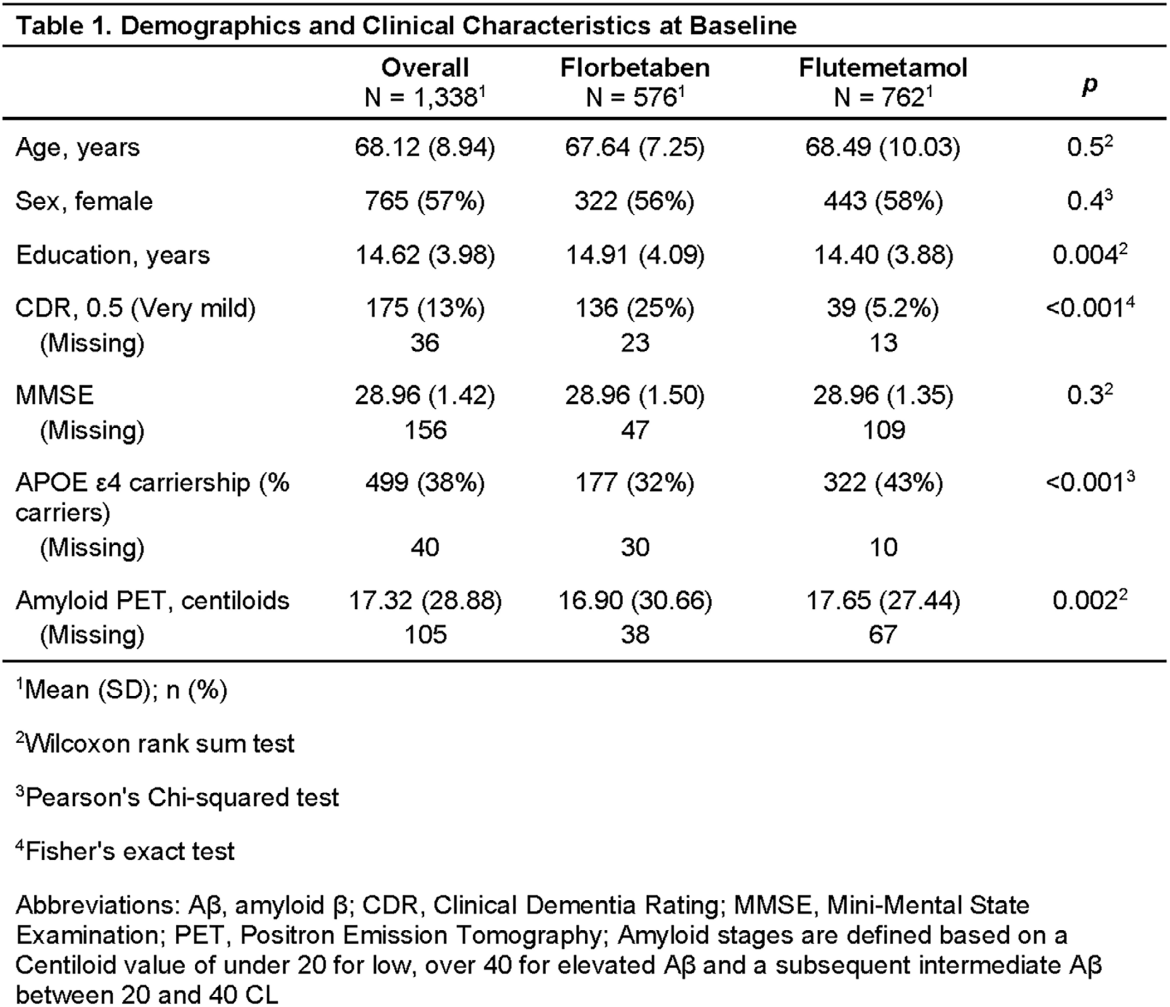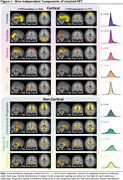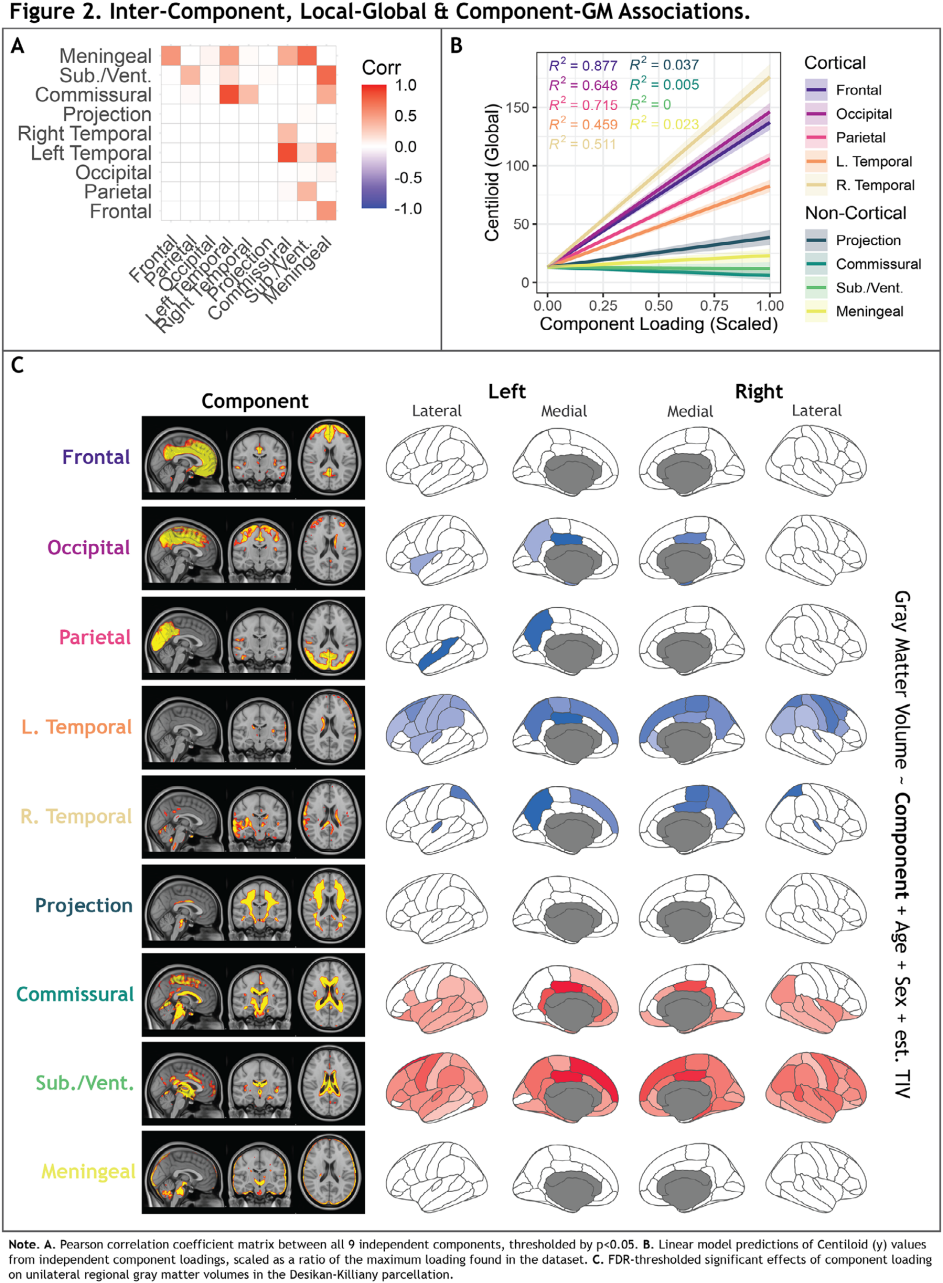# Capturing Alzheimer's Disease Heterogeneity With Spatially Independent Patterns of Amyloid Accumulation

**DOI:** 10.1002/alz70856_099751

**Published:** 2025-12-25

**Authors:** Leonard Pieperhoff, Luigi Lorenzini, Mario Tranfa, Prithvi Arunachalam, Craig Ritchie, Mercè Boada, Marta Marquié, Wiesje M. van der Flier, Rik Vandenberghe, Bernard J Hanseeuw, Pablo Martínez‐Lage, Pierre Payoux, Pieter Jelle Visser, Michael Schöll, Giovanni B. Frisoni, Andrew W. Stephens, Christopher Buckley, Gill Farrar, Frank Jessen, Juan Domingo Gispert, Henk Mutsaerts, Tiago Gil Oliveira, Lyduine E. Collij, Frederik Barkhof, Alle Meije Wink, AMYPAD consortium

**Affiliations:** ^1^ Amsterdam University Medical Center (Amsterdam UMC), Amsterdam, North Holland, Netherlands; ^2^ University of Naples Federico II, Naples, Italy; ^3^ Scottish Brain Sciences, Edinburgh, Scotland, United Kingdom; ^4^ Ace Alzheimer Center Barcelona – International University of Catalunya (UIC), Barcelona, Spain; ^5^ Ace Alzheimer Centre Barcelona ‐ Universitat Internacional de Catalunya, Barcelona, Spain; ^6^ Alzheimer Center Amsterdam, Neurology, Vrije Universiteit Amsterdam, Amsterdam UMC, Amsterdam, Netherlands; ^7^ KU Leuven, Leuven, Belgium; ^8^ Institute of Neuroscience, UCLouvain, Brussels, Belgium; ^9^ Fundación CITA‐Alzhéimer Fundazioa, Donostia‐San Sebastian, Spain; ^10^ Université de Toulouse, Toulouse, France; ^11^ Alzheimer Center Limburg, Mental Health and Neuroscience Research Institute, Maastricht University, Maastricht, Netherlands; ^12^ Alzheimer Center Amsterdam, Department of Neurology, Vrije Universiteit Amsterdam, Amsterdam UMC location VUmc, Amsterdam, Netherlands; ^13^ University of Gothenburg, Gothenburg, Västra Götalands län, Sweden; ^14^ Geneva Memory Center, Department of Rehabilitation and Geriatrics, Geneva University Hospitals, Geneva, Geneva, Switzerland; ^15^ Life Molecular Imaging GmbH, Berlin, Germany; ^16^ GE HealthCare, Chalfont St Giles, Buckinghamshire, United Kingdom; ^17^ University Hospital Cologne, Cologne, Germany; ^18^ Barcelonaβeta Brain Research Center (BBRC), Pasqual Maragall Foundation, Barcelona, Spain; ^19^ School of Medicine, Institute of Life and Health Sciences (ICVS), University of Minho, Braga, Portugal; ^20^ Clinical Memory Research Unit, Department of Clinical Sciences Malmö, Faculty of Medicine, Lund University, Lund, Sweden; ^21^ University College London, London, United Kingdom

## Abstract

**Background:**

Current Alzheimer's disease (AD) research and clinical trials generally assume a single trajectory of cortical amyloid beta (Aβ) accumulation, but amyloid positron emission tomography (PET) studies suggest multiple trajectories. We aimed to better characterize AD heterogeneity using independent spatial component analysis of amyloid‐PET.

**Methods:**

We spatially decomposed amyloid‐PET scans obtained with two radiotracers [^18^F]Flutemetamol (*n* = 772) and [^18^F]Florbetaben (*n* = 568) from the AMYPAD Prognostic & Natural History Study, a pan‐European multi‐site study of non‐demented older individuals (CDR < 1, age ≥ 50). Using FSL MELODIC, we ran n‐dimensional spatial group‐level (range_n_=1‐15) independent component analysis (gICA) on MNI152‐registered SUVR images (using the whole cerebellum as reference), independently for each radiotracer. The final gICA dimensionality was chosen by optimizing for the largest number of spatial cross‐correlations (R>0.6) between radiotracers. Component loading was estimated via spatial regression of the resulting components on the individual amyloid‐PET scans. We then investigated how the resulting components relate to each other (Pearson correlations), to global Aβ burden measure with Centiloid (linear models), and to regional GM volumes independently of age, sex and estimated intracranial volume (linear models).

**Results:**

Subject characteristics at baseline can be found in Table 1. A 9‐dimensional gICA robustly identified five cortical components‐ frontal, parietal, occipital, unilateral left and right temporal cortices and additionally four non‐cortical components encompassing projection fibers, commissural fibers, lateral ventricular borders, and meninges (Figure 1). All components were largely independent of each other, only the meningeal component correlated to other components and the commissural component correlated to the temporal cortical components (Figure 2A). The strongest positive associations with Centiloid were found for the cortical components (R^2^_range_=0.46‐0.88), while non‐cortical components were only sparsely associated (R^2^_range_=0‐0.04; Figure 2B). GM volume was, independently of age, sex, and estimated intracranial volume, negatively associated to cortical component loading most prominently with left and right temporal loading, while wide‐scale positive associations with GM volume were found for the commissural component and the subcortical/ventricular component (Figure 2C).

**Conclusions:**

We found five cortical and four non‐cortical independent components of amyloid‐PET signal with high consistency between two radiotracers and which related differentially to GM volumes. This method allowed us to quantify several features that could capture heterogeneous disease processes.